# A new species of *Rhopalosiphum* (Hemiptera, Aphididae) on *Chusquea tomentosa* (Poaceae, Bambusoideae) from Costa Rica

**DOI:** 10.3897/zookeys.166.2387

**Published:** 2012-01-20

**Authors:** Nicolás Pérez Hidalgo, David Martínez-Torres, Jorge Mariano Collantes-Alegre, William Villalobos Muller, Juan M. Nieto Nafría

**Affiliations:** 1 Departamento de Biodiversidad y Gestión Ambiental, Universidad de León, E-24071, León, Spain; 2 Institut Cavanilles de Biodiversitat i Biologia Evolutiva, Universitat de València, E-46071, Valencia, Spain; 3 Centro de Investigación en Biología Celular y Molecular, Universidad de Costa Rica, 11501-2060, San José, Costa Rica

**Keywords:** *Rhopalosiphum*, aphids, new species, molecular, Costa Rica

## Abstract

The new species *Rhopalosiphum chusqueae* Pérez Hidalgo & Villalobos Muller, is described from apterous viviparous females caught on *Chusquea tomentosa* in Cerro de la Muerte (Costa Rica). The identity of the species is supported both by the morphological features and by a molecular phylogenetic analysis based on a fragment of the mitochondrial DNA containing the 5’ region of the cytochrome c oxidase 1 (COI) and on the nuclear gene coding for the Elongation factor-1 alpha (EF1α). The taxonomic position of the new species is discussed. An identification key to the Aphidinae species living on plants of Bambusoideae (Poaceae) is presented.

## Introduction

The high diversity of organisms in Costa Rica has been referred to as a product of diverse ecosystems resulting from the interaction between complex microclimates, soils, topography, and a variety of biological processes, as well as the position of the country in the land-bridge between North and South America. Costa Rica’s biodiversity comprises more than 500,000 species of organisms, approximately 84% of which are yet to be described. This percentage is even higher (90%) if we take insects, fungi, bacteria and viruses into account (Sánchez-Azofeifa et al. 2001). As for the number of aphid species present in Costa Rica, the list was recently extended (Pérez Hidalgo et al. 2009; Zamora Mejías et al. 2010; Villalobos Muller et al. 2010) and research is ongoing.

**Figure 1.**
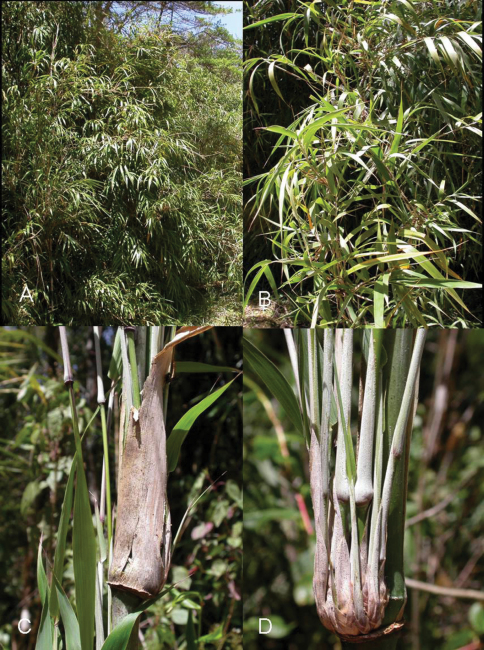
**A**View of the area where the types of *Rhopalosipum chusqueae* were captured, in Cerro de la Muerte (Costa Rica), with *Chusquea tomentosa* **B** view of the plant **C** and **D** details of the area where the aphids were located.

During an expedition in 2008 in the area of Cerro de la Muerte (Cordillera de Talamanca), Costa Rica, three apterous viviparous females and several nymphs were collected on *Chusquea tomentosa* (Fig. 1). At first, they were assigned to the subtribe Rhopalosiphina Mordvilko, 1914 (Aphidini Latreille, 1802). This identification was confirmed in the laboratory when it was verified that the marginal papillae on abdominal segments I and VIII were in dorsal position to the corresponding stigmata. The morphological characters of the specimens resembled those of the genus *Rhopalosiphum* Koch, 1854, though the length of the setae were reminiscent of species in the subgenus *Paraschizaphis* Hille Ris Lambers, 1947 (*Schizaphis* Börner, 1931).

According to Valenzuela et al. (2009), *Rhopalosiphum* and *Schizaphis* form a monophyletic group with *Melanaphis* van der Goot, 1917, the separation between them being unclear. The vein structure of the wings separates *Schizaphis* from *Rhopalosiphum*, however, as there were no alates available, molecular analyses were carried out to verify the relationship with the genus *Rhopalosiphum* through qualitative and quantitative characters. Molecular analyses are normally used to determine species and resolve taxonomic problems in the family Aphididae (Lozier et al. 2008; Foottit et al. 2008, 2009; Lee et al. 2010; Pike et al. 2010).

A microscopic morphotaxonomic study of the specimens enabled the hypothesis that they could not be assigned to any known species, and strengthened the hypothesis that they be assigned to *Rhopalosiphum*. Molecular phylogenetic analysis based (1) on a fragment of the mitochondrial DNA containing the 5’ region of the cytochrome c oxidase 1 (COI) and (2) on the nuclear gene coding for the Elongation factor-1 alpha (EF1α) were used to verify both hypotheses.

Several species included in the subfamily Aphidinae are known living on plant species of the subfamily Bambusoideae (Poaceae), but only two belong to *Rhopalosiphum* (Aphidini): *Rhopalosiphum arundinariae* (Tissot, 1933) and *Rhopalosiphum rufiabdominale* (Schrank, 1899), and none to the genus *Schizaphis*.

## Material and methods

### Material studied

Three apterous viviparous female and several nymphs (sample CRI-235) were recorded on *Chusquea tomentosa* Y. Widmer et L. G. Clark (Poaceae: Bambusoideae: Bambuseae: Chusquinae) in Ojo de Agua (Cerro de la Muerte, Cordillera de Talamanca, Costa Rica) (9°36'N, 83°47'W), 2968 m, 26.ii.2008.

### Morphological study

Thirty-three quantitative characteristics and the qualitative features of shape, sclerotization, pigmentation and cuticular ornamentation, were considered. The method used for measurements is that normally employed in our studies (Nieto Nafría and Mier Durante 1998). A camera lucida fitted to the microscope was used for the drawings and the microphotographs were taken with a Leica DC digital camera with IM 1000 version 1.10 software.

### DNA extraction and PCR amplification

Total DNA was extracted separately from two samples, one of them containing a single nymph and the second the contents of the abdomen of 3 apterous adults, all kept in 96% ethanol. We followed the HotSHOT (Hot Sodium Hydroxide and Tris) method (Truett et al. 2000).

PCR amplification of the two gene fragments analyzed was carried out on 3 µl of the extracted DNA. A 710 bp fragment of the 5’ region of the mitochondrial cytochrome c oxidase subunit 1 (COI) was amplified using primers LCO1490 and HCO2198, described by Folmer et al. (1994). PCR conditions for COI amplification were as follows: 94°C for 1 min; 35 cycles of 94°C for 30 s, 48°C for 1 min and 68°C for 1 min; a final extension step of 7 min at 68°C was included after cycling. Amplification of the Elongation factor-1 alpha (EF1α) gene fragment was performed using two consecutive PCR reactions with primers Efs175 (Moran et al. 1999) and Efr1 (5’GTGTGGCAATSCAANACNGGAGT3’) in the first reaction and then primers Efs175 and Efr2 (5’TTGGAAATTTGACCNGGGTGRTT3’) in the second hemi-nested reaction. PCR conditions used in the first reaction were: 94°C for 1 min; 40 cycles of 94°C for 30 s, 50°C for 1 min and 68°C for 1.5 min; a final extension step of 7 min at 68°C was included after cycling. The hemi-nested PCR was done similarly but using 52°C for the annealing step and using 1 µl of the first PCR product.

### Sequencing and analysis of DNA sequences

PCR products were purified by ammonium precipitation and reconstituted in 10 μL of LTE buffer (10mM Tris, 0,1mM EDTA). Direct sequencing of amplified fragments was done in both directions using PCR primers (Efr2 was used as reverse primer for sequencing the EF1α fragment). Sequencing was conducted using the Big Dye Terminator v3.1 Cycle Sequencing Kit (Applied Biosystems) following the manufacturer’s instructions, and samples were loaded onto an ABI 3700 automated sequencer.

Chromatograms were revised and sequences corresponding to each sample assembled using the Staden package v1.6.0 (Staden et al. 2000). Multiple alignments were carried out with Clustal X v1.81 (Thompson et al. 2002) with gap opening and gap extension penalties of 10.0 and 0.2, respectively, and subsequently manually revised.

Phylogenetic analysis of COI sequences were done using *MEGA* version 4 (Tamura et al. 2007). For EF1α sequences ModelTest (Posada and Crandall 1998) was used to find the evolutionary model that best fitted sequence data and phylogenetic reconstruction was done using RAxML (Stamatakis et al. 2008).

## Results

### Morphological data

**Figure 2.**
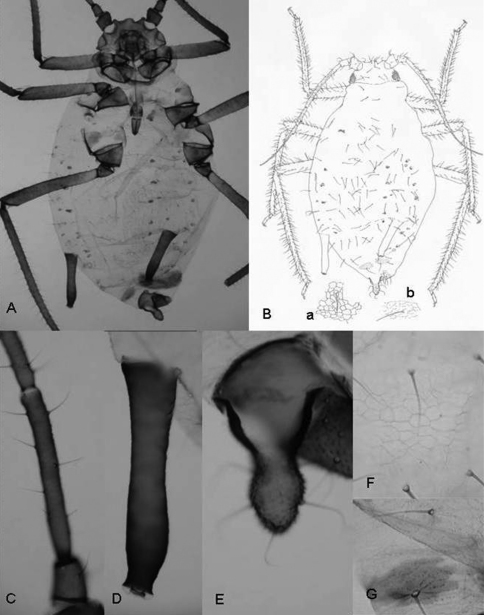
*Rhopalosiphum chusqueae* sp. n. **A, B** *Habitus* **C** antennal segment III **D** siphunculus **E** cauda. **F** (and **B-a**) detail of the cuticule of abdominal segment 3 **G** (and **B-b**) detail of cuticule of abdominal segment 8.

**Figure 3.**
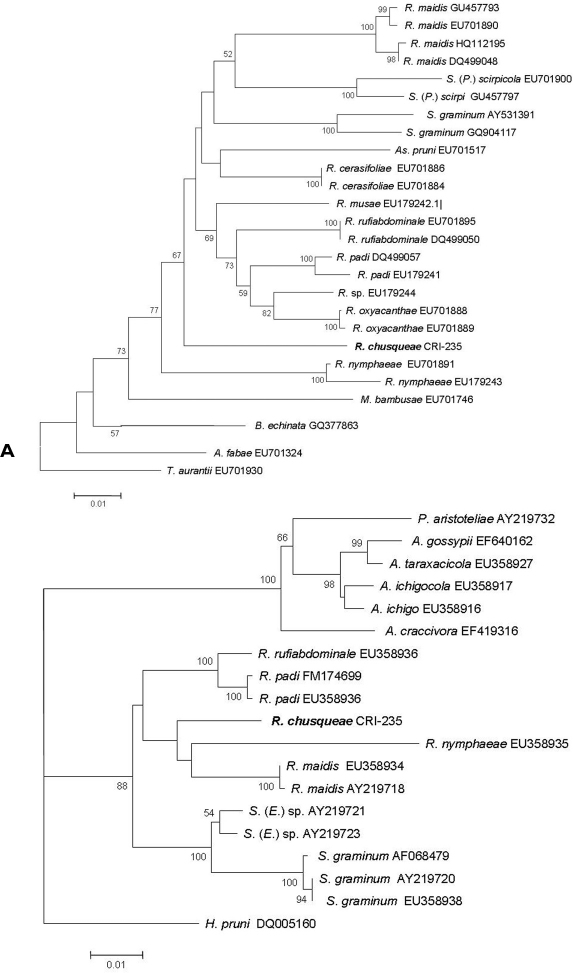
**A** Neighbour joining tree based on Kimura 2P distances obtained for the COI sequences from our new species (*Rhopalosiphum chusqueae*) and different Aphidini representatives obtained from the NCBI database. **B** Maximum Likelihood tree obtained for the EF1α sequences for our new species (*Rhopalosiphum chusqueae*) and different Aphidini representatives obtained from the NCBI database. Bootstrap support values obtained after 1000 replicates in A and 100 in B are indicated above branches if higher than 50%. Initials for genera are as follows: A, *Aphis*; As, *Asiphonaphis*; B, *Braggia*; H, *Hyalopterus*, M, *Melanaphis*; P, *Paradoxaphis*; R, *Rhopalosiphum*; S, *Schizaphis* (*Schizaphis*); S. (E.), *Schizaphis* (*Euschizaphis*); S. (P.), *Schizaphis* (*Paraschizaphis*); T, *Toxoptera*.

A study of the qualitative and quantitative (metric and meristic) characters of the specimens enabled us to establish the hypothesis that they belong to the genus *Rhopalosiphum* as, apart from the above-mentioned character of the marginal papillae on the abdomen, (1) when alive they are ovoid and when preserved the body is not very long and the margins are curved (Figs 2A, 2B), (2) the dorsal cuticle of the thorax and abdomen is membranous, except for the presence of intersegmental sclerites and a pair of large sclerites on abdominal segment VIII (Figs 2A, 2B), (3) the dorsal cuticle has a more or less regular reticulate area formed by coalescent spinules (Figs 2Ba, 2Bb, 2F, 2G), (4) the siphunculi are longer than the cauda and clearly constricted underneath the apical edge (Figs 2A, 2B, 2D), and (5) there are few setae on the cauda.

A comparison of the characters of these specimens with those of apterae in other species of *Rhopalosiphum* and *Schizaphis* also strengthened the hypothesis that they could not be assigned to any known species.

### Molecular data

A 710 bp DNA fragment containing a portion of the mitochondrial COI gene was amplified through PCR from the two samples analysed. Useful sequences obtained from each sample consisted of 658 nucleotides. Identical sequences were obtained for both samples so that a single sequence was finally assigned and deposited in Genbank (accession number HE604204). The online identification engine available at the Barcode of Life Data Systems (BOLD) (Ratnasingham and Hebert 2007) using the COI species database, failed to find any record corresponding to any identified species that matched our sequence. After a BLASTN search against the non-redundant nucleotide database at the NCBI, sequences from different *Rhopalosiphum* species were most similar to our sequence (93–94% identical) followed by *Schizaphis* sequences (92–93% identical). We then aligned our sequence with sequences from all *Rhopalosiphum* species available at the NCBI database and from species representative of closely related genera (*Schizaphis*, *Melanaphis*, etc.) that we had previously retrieved from the database, and built a phylogenetic tree (Fig. 3A). The tree shows that the sequence from our unknown species groups with relatively high support within a monophyletic clade that contains all other *Rhopalosiphum* and *Schizaphis* COI sequences occupying a rather basal position within that clade.

For the Elongation factor-1 alpha (EF1α) gene fragment, we obtained an identical sequence from the two analyzed samples of 987 bp which was deposited in the Genbank with accession number HE604205. Using sequences available for EF1α in NCBI for different *Rhopalosiphum* and closely related species within Aphidini, an ML tree was built that included the sequence obtained for our unknown species (Fig. 3B). As with the COI sequence, our unknown species grouped with strong support within a monophyletic clade that also included sequences from *Schizaphis* and *Euschizaphis*. However, unlike the COI tree, both *Rhopalosiphum* and *Schizaphis*-related sequences separated into two distinct clades, though with very low bootstrap support.

## Discussion and conclusion

Molecular data using both mitochondrial COI and nuclear EF1α gene sequences confirmed that the *Rhopalosiphum chusqueae* specimens belong to the same monophyletic clade as other *Rhopalosiphum* species occupying a rather basal position in the group likely closely related to other divergent *Rhopalosiphum* species such as *Rhopalosiphum nymphaeae*. Both trees revealed the close relationship between *Rhopalosiphum* and *Schizaphis* genera. Although COI sequences are widely used in taxonomy, their utility for phylogeny reconstructions seems rather limited as their phylogenetic signal is somewhat weak in comparison with other markers (Wilson 2010). Contrarily, EF1α is widely used in phylogenetic reconstructions and its use in insect phylogeny has been shown to be informative (Simon et al. 2009; Wilson 2010). In this respect, although the COI analysis did not recover the monophyly of *Rhopalosiphum* and *Schizaphis* genera separately, ML analysis of EF1α separated both genera and clearly *Rhopalosiphum chusqueae* grouped within the *Rhopalosiphum* clade, which, along with our morphometric data discussed above, supports its assignation to the *Rhopalosiphum* genus.

Approximately 15 species are classified in the genus *Rhopalosiphum* (Remaudière and Remaudière 1997; Zhang and Qiao 1997; Eastop and Blackman 2005; Blackman and Eastop 2006) associated with arboreal Rosaceae (*Prunus* or Pyroidea) as the primary host and with Poaceae, Cyperaceae or, less frequently, other plants as the secondary host if their cycle is dioecious, or only with one of them if their cycle is monoecious. Most of the species probably originate in North America, with a subsidiary centre of dispersal in Central Asia (Blackman and Eastop 1994; Halbert and Voegtlin 1998; Blackman and Eastop 2006). Five of its species have an exclusively Nearctic distribution: *Rhopalosiphum arundinariae* (Tissot), *Rhopalosiphum cerasifoliae* (Fitch), *Rhopalosiphum enigmae* Hottes & Frison, *Rhopalosiphum laconae* Taber, *Rhopalosiphum nigrum* Richards, and *Rhopalosiphum padiformis* Richards; and another four Nearctic species have been introduced in other parts of the world: *Rhopalosiphum parvae* Hottes & Frison and *Rhopalosiphum rufulum* Richards in Europe, *Rhopalosiphum musae* Schouteden has been recorded in areas of Europe, Central Asia, Africa and Australia, and *Rhopalosiphum oxyacanthae* (Schrank) is known in Central- and South-America, Europe, Asia and Australia. To date, only four species, linked mainly to crops, have been recorded in Central American countries: *Rhopalosiphum maidis* (throughout Central America), *Rhopalosiphum nymphaeae* in Panama, *Rhopalosiphum padi* in Costa Rica and Panama, and *Rhopalosiphum rufiabdominale* in Honduras, Costa Rica and Panama (Evans and Halbert 2007; Quirós et al. 2009; Villalobos Muller et al. 2010); *Rhopalosiphum oxyacanthae* is also known in Central America, without country (Blackman and Eastop 2006).

Species of *Rhopalosiphum* most resembling the new species due to their morphological characters are *Rhopalosiphum rufiabdominale* and *Rhopalosiphum padiformis*. The former originated from East Asia (Blackman and Eastop 2006) and is currently widely distributed. *Rhopalosiphum padiformis* originates from North America. *Rhopalosiphum chusqueae* sp. nov. coincides with both species in the length of the setae, with *Rhopalosiphum padiformis* in the number of antennal segments and shape of the cauda, and with specimens of *Rhopalosiphum rufiabdominale* in the 4 setae on abdominal segment VIII (*Rhopalosiphum rufiabdominale* has 3 to 8 setae on this segment). It is easily distinguished from them because the antennae in *Rhopalosiphum rufiabdominale* are five-segmented and the dorsal setae in *Rhopalosiphum padiformis* are not pointed and abdominal segment VIII only has 2 setae. Bamboo species are the host plants of the mentioned *Rhopalosiphum rufiabdominale* and *Rhopalosiphum arundinariae*; this last species can be easily differentiated from *Rhopalosiphum chusqueae* by the shape of the cauda (short and more or less triangular or rounded) and siphunculus (more or less tapering) and by much shorter setae on body dorsum and appendages.

In view of the above, a new species can be established, the description of which follows.

### Rhopalosiphum chusqueae

Pérez Hidalgo & Villalobos Muller sp. n.

urn:lsid:zoobank.org:act:D3A0466B-3858-46A8-A4BB-4618BDD956D3

http://species-id.net/wiki/Rhopalosiphum_chusqueae

#### Holotype.

Apterous viviparous female number 1 of measurement series, caught on *Chusquea tomentosa*, Pérez Hidalgo & Villalobos Muller *leg*., deposited in the Aphidological Collection of the University of León (CZULE), sample CRI-235.

#### Paratypes.

2 apterous viviparous females (in separated slides) caught with the holotype.

#### Etymology.

The specific epithet, *chusqueae* is the genitive singular of the generic name of the aphid’s host plant.

#### Apterous viviparous females

(Figure 2). When alive globular oval and brown with white spots of wax on abdomen. Mounted 2.20–2.72 mm and pale in general with head, antennae, legs, siphunculi and cauda dark-brown.

Antennae 0.63–0.79 times body length. Antennal segment III (0.32–0.43 mm) shorter than segment IV (0.21–0.25 mm) plus V (0.20–0.26 mm); with setae 55–65 μm long and 1.8–2.6 times the articular diameter of the segment. Terminal process of segment VI (0.44–0.47 mm) 3.9–4.4 times the base (0.32–0.43 mm). Rostrum 0.52–0.61 mm long, reaching middle coxae, 0.19–0.27 times the body length. Ultimate rostral segment 0.13–0.15 mm long, approximately 1.7 times its basal width and 1.1 second segment of hind tarsus; it carries two accessory setae. Marginal papillae present on prothorax, on the abdominal segment 1 and 7, which are dorsally placed to the respective spiracles, and sometimes on segments 3 and 6. Dorsum of the abdomen with spinules forming reticulate ornamentation. Dorsal setae on abdominal segment 3 with delicate, pointed and 25–30 μm long and 3.0–3.9 times the articular diameter of antennal segment III and shorter than ventral ones, which are 90–110 μm long. Siphunculi slightly swollen with marked narrowing below the flange, 0.41–0.45 mm long, 0.16–0.20 times the body length and 2.1–2.2 times cauda. Abdominal segment 8 with two sclerites and four setae 90–110 μm long, delicate and pointed. Genital plate with 2 discal setae and near 26 posterior ones. Cauda finger-like, 0.19–0.21 mm long and carrying 5 setae.

#### Distribution and host-plant.

*Chusquea tomentosa* (Poaceae, Bambusoideae) is the only known host of *Rhopalosiphum chusqueae*. This bamboo is endemic to the country and can be found in several areas of the Cordillera de Talamanca at an altitude of between 2450 and 3000 m (Widmer 1997; Hammel et al. 2003). Species of *Chusquea* (approximately 120 described) can be found at between 800 and 3800 m in dry and humid forests from Mexico to Chile and Argentina (Clark 1989). As species in the genus *Rhopalosiphum* are not strictly stenophagous, *Rhopalosiphum chusqueae* may also live on other species of *Chusquea*, or even on other bamboos and live in other parts of America.

On the plant, the aphids live close to the nodes well protected by the leaves (Figs 1C, 1D) and not easily detectable, as shown by fruitless efforts to locate other colonies.

So far, only one aphid species had been recorded on *Chusquea*: *Hysteroneura setariae* (Thomas) on *Chusquea abietifolia* Griseb, in Cuba (Holman 1974).

Blackman and Eastop (1994) present two identification keys to the aphid species living on *Arundinaria* and on *Bambusa*, genera that include arboreal bamboos; several of these aphid species belong to the subfamily Aphidinae. Blackman and Eastop (2006) report the presence of aphid species on several genera of non-arboreal bamboos such as *Chusquea*, *Pseudosasa*, *Sinoarundinaria*, *Thamnocalamus*, *Thysanolaena*, and also *Arundinaria* (other bamboo genera are included but no Aphidinae species have been recorded on them), and for the identification of these species the reader is forwarded to the “keys [of 1994] to aphids on *Arundinaria* and *Bambusa*”, or to the “keys [of 2006] to apterae on *Digitaria* and other genera of herbaceous Poaceae”. To make the identification work easier, it seems useful to present one compendium-key to the identification of apterous viviparous females of Aphidinae species recorded on species of Bambusoideae in the World.

This key has been prepared using the general structure and several couplets in all of those keys by Blackman and Eastop; thirteen Aphidinae species and subspecies have been included, and are: *Hysteroneura setariae* (Thomas, 1878), *Melanaphis arundinariae* (Takahashi, 1937), *Melanaphis bambusae* (Fullaway, 1910), *Melanaphis meghalayensis bengalensis* Raychaudhuri [D.N.] and Banerjee [C.], 1974, *Melanaphis meghalayensis meghalayensis* Raychaudhuri [D.N.] and Banerjee [C.], 1974, *Melanaphis pahanensis* (Takahashi, 1950), *Melanaphis sacchari* (Zehntner, 1897), *Rhopalosiphum arundinariae* (Tissot, 1933) and *Rhopalosiphum rufiabdominale* (Schrank, 1899) (Aphidinae Aphidini Rhopalosiphina), and *Sitobion bambusicola* (Ghosh [L.K.], 1986), *Sitobion fragariae* (Walker, 1848), *Sitobion miscanthi* (Takahashi, 1921) and *Sitobion papillatum subnudum* Remaudière, 1985 (Aphidinae Macrosiphini).

**Table:** 

1	Siphunculus without apical zone of polygonal reticulation. Abdominal segments I and VII with marginal tubercles (papillae) placed dorsally to the respective spiracular apertures. Cuticle of dorsum of the abdomen membranous, a sclerotized patch absent	2
–	Siphunculus with apical zone of polygonal reticulation (at least two rows of cells). Abdominal segments I and VII usually without marginal tubercles (papillae), but if they are present then spinal papillae present on head and several abdominal segments. Dorsum of the abdomen with a sclerotized patch more or less extended and pigmented	12
2	Aphids spindle-shaped, green when alive. Siphunculus very small (less than 0.7 times cauda), thin, cylindrical and narrow-based, flangeless, and with not functional aperture	*Hyalopterus pruni* [and other *Hyalopterus* spp.]
–	Aphids broad oval-shaped. Siphunculus 0.5–2.5 times cauda (if less than 0.6 times then less than 2 times longer than its basal width), shaped differently and with functional aperture	3
3	Siphunculus short, usually thick or rather thick, less than (often much less than) 2.4 times longer than its basal width, 0.4–1.2 times cauda, and usually with a well-developed, rather swollen flange	4
–	Siphunculus usually longer than cauda (if less than 1.2 times cauda then it is more than 2.4 times its basal width and/or has a small flange), tapering, cylindrical or swollen	9
4	Setae on antennal segment III at most 1.5 times the basal diameter of the segment. [Alatae viviparous females with wing veins dark bordered]	5
–	Setae on antennal segment III at least 2.0 times the basal diameter of the segment. [Alatae viviparous females with wing veins not dark bordered]	6
5	Cauda with only 4-6 setae. Coxae dark	*Melanaphis bambusae*
–	Cauda with 7-20 setae. Coxae pale	*Melanaphis sacchari*
6	Antennae five-segmented. Siphunculus 1.5 times its basal width at least	*Melanaphis arundinariae*
–	Antennae six-segmented. Siphunculus 1.4 times its basal width at most	7
7	Siphunculus 1.1–1.4 times its basal width. Terminal processus of antennal segment VI at most 2.3 times the base	*Melanaphis pahanensis*
–	Siphunculus 0.8–0.9 times its basal width. Terminal processus of antennal segment VI at least 2.2 times the base	[*Melanaphis meghalayensis*] 8
8	Cauda with 4–6 setae and anterior half of the genital plate with 4–7 setae	*Melanaphis meghalayensis meghalayensis*
–	Cauda with 7–10 setae and anterior half of the genital plate with 2 setae	*Melanaphis meghalayensis bengalensis*
9	Setae on antennal segment III shorter than the basal width of the segment	10
–	Setae on antennal segment III longer than the basal width of the segment	11
10	Cauda at least 1.5 times its basal width, finger-shaped, with basal constriction, paler than cauda, and usually with 4 setae. [Alate viviparous females with only one oblique vein in hindwing]	*Hysteroneura setariae*
–	Cauda a little longer that its basal width, cone-shaped, without basal constriction, as dark as siphunculi, and with approximately 8 setae. [Alate viviparous females with two oblique veins in hindwing]	*Rhopalosiphum arundinariae*
11	Antennae usually five-segmented. Setae on antennal segment III 3.0–5.0 times the basal width of the segment. Abdominal segment VIII with 3–8 setae. Ultimate rostral segment 1.3–1.8 times second segment of the hind tarsus. Terminal processus of antennal segment VI 4.0–6.5 times the base	*Rhopalosiphum rufiabdominale*
–	Antennae six-segmented. Setae on antennal segment III 1.8–2.6 times the basal width of the segment. Abdominal segment VIII with 4 setae. Terminal processus of antennal segment VI 3.9-–4.4 times the base	*Rhopalosiphum chusqueae* sp. n.
12	Spinal tubercles (papillae) present on the head and abdominal segments (V)VI-VIII; marginal ones present on prothorax and abdominal segments (I)II–V and infrequently on VII	*Sitobion papillatum subnudum*
–	Spinal tubercles (papillae) absent; marginal ones on abdominal segment II–V usually absent, and always absent on abdominal segments I and VII	13
13	Cauda dusky (but not as dark as siphunculi) and with a rather pointed apex. Siphunculus 2.0–2.1 times cauda. Aphids yellowish when alive	*Sitobion bambusicola*
–	Cauda pale (very contrasted with siphunculi) with a variably shaped apex. Siphunculus 1.4–2.7 times cauda. Aphids variable in colour when alive	14
14	Siphunculi 1.75–2.25 times cauda, which has a rather rounded apex	*Sitobion fragariae*
–	Siphunculi 1.4–1.9 times cauda, which has a rather pointed apex	*Sitobion miscanthi*

## Supplementary Material

XML Treatment for Rhopalosiphum chusqueae
